# Integration of Polylactide into Polyethylenimine Facilitates the Safe and Effective Intracellular siRNA Delivery

**DOI:** 10.3390/polym12020445

**Published:** 2020-02-14

**Authors:** Guo-Bin Ding, Xue Meng, Peng Yang, Binchun Li, Roland H Stauber, Zhuoyu Li

**Affiliations:** 1 Institute of Biotechnology, the Key Laboratory of Chemical Biology and Molecular Engineering of Ministry of Education, Shanxi University, Taiyuan 030006, China; meng.0923@foxmail.com (X.M.); peng114011@sxu.edu.cn (P.Y.); libinchun@sxu.edu.cn (B.L.); rstauber@uni-mainz.de (R.H.S.); 2 Institutes of Biomedical Sciences, Shanxi University, Taiyuan 030006, China; 3 Molecular and Cellular Oncology, University Medical Center Mainz, Langenbeckstrasse 1, 55101 Mainz, Germany

**Keywords:** PEI-PLA copolymer, siRNA targeting PKM2, serum stability, cytotoxicity, transfection efficiency

## Abstract

Polyethylenimine (PEI) is a gold standard polymer with excellent transfection efficacy, yet its severe toxicity and nondegradability hinders its therapeutic application as a gene delivery vector. To tackle this problem, herein we incorporated the biodegradable polylactide (PLA) into the branched PEI by synthesizing a PEI-PLA copolymer via a facile synthetic route. PLA modification significantly improved the cytocompatibility of PEI, PEI-PLA copolymer showed much higher cell viability than PEI as verified in three different human cancer cell lines (HCT116, HepG2 and SKOV3). Interestingly, the PEI-PLA copolymer could effectively bind siRNA targeting PKM2, and the obtained polyplex displayed much higher stability in serum than naked siRNA as determined by agarose gel electrophoresis. Moreover, cellular uptake study demonstrated that PEI-PLA could efficiently deliver the Cy5-labled siRNA into the three tested cancer cell lines, and the transfection efficiency is equivalent to the commercial Lipofectamine^®^ 2000. Finally, it is noteworthy that the polyplex is comparable to Lipo2000 in down-regulating the expression of PKM2 at both mRNA and protein level as measured by q-PCR and western blotting, respectively. Overall, the PEI-PLA copolymer developed in this study has the potential to be developed as a versatile carrier for safe and effective delivery of other nucleic acid-based agents.

## 1. Introduction

Cancer is still the leading cause of mortality worldwide, and conventional chemotherapy and radiotherapy suffer from the disadvantages of low efficacy and adverse systemic toxicity to normal tissues [1,2]. Nucleic acid-based gene therapy opens new opportunities for cancer treatment and has great potential in preventing the deaths of cancer patients [3]. Small interfering RNAs (siRNAs) are a kind of artificial double-stranded macromolecules with a length of 19–21 nt that have an extremely high specificity to their complementary mRNAs and only trigger the degradation of the target mRNAs [4]. Thus, siRNA-based RNA interference (RNAi) offers an invaluable technique for personalized cancer therapy, as it can selectively and effectively knockdown the expression of targeted genes [5,6]. Nevertheless, there are still some obstacles need to be overcome during the development of siRNA-based drugs, including the poor cellular internalization due to the large molecular mass (~13 kDa) and electronegativity of siRNAs, and the instability of siRNAs in blood circulation because of the presence of endonuclease, which greatly hinders the translation of siRNAs from bench to clinical settings [4,7,8].

In the past few decades, various delivery systems have been developed to improve the intracellular trafficking of siRNAs. Although viral vectors exhibit excellent efficiency in gene transfer, there are fundamental drawbacks, including serious safety concerns due to their undesirable off-target effects (immunogenicity, carcinogenesis, inflammatory response and toxicity) and the difficulty in large-scale production [9,10]. Therefore, nonviral vectors have been extensively investigated as alternative gene delivery systems due to their good biocompatibility and low toxicity compared to viral vectors. To date, a variety of nonviral vectors including peptides-based delivery carriers, lipid-based delivery vectors, polymer-based delivery systems, inorganic nanoparticles-based vectors and stimuli-sensitive nanovehicles have been explored by researchers for effective siRNA delivery [4,11,12,13,14,15,16,17,18]. Among the above-mentioned siRNA delivery systems, studies on cationic polymers are gaining increasing attention due to their strong capability to condense large siRNAs and protect siRNAs against ribozyme degradation [19]. As a gold standard polymer with high transfection efficacy, polyethylenimine (PEI), especially branched PEI (bPEI), is widely employed for in vitro and in vivo siRNA delivery due to its well-known proton sponge effect, which is responsible for efficient endosome escape [20,21,22]. Unfortunately, the practical application of PEI for gene therapy is far from satisfactory, owing to its molecular weight-dependent cytotoxicity, nondegradability and aggregation of polyplexes during blood circulation [20]. As a result, numerous efforts have been undertaken to modify PEI with various biocompatible fragments with the aim of improving its biocompatibility and stability, while mitigating its toxicity [23,24,25,26,27]. As we know, there are only a few reports on the integration of polylactide (PLA) into PEI [28,29], although the biodegradable PLA is frequently used in drug delivery systems and has been approved by FDA for medical use [30].

Pyruvate kinase is a key rate-limiting enzyme in glycolysis that catalyzes the conversion of phosphoenolpyruvate (PEP) and ADP to pyruvate and ATP [31]. There are four pyruvate kinase isoforms expressed in different mammalian cells and tissues, named PKL, PKR, PKM1 and PKM2, [32]. Among the four pyruvate kinase isoenzymes, PKM2 is preferentially expressed in most cancer cells, which makes it an attractive target for tumor therapy [33]. Accumulating evidence reveals that knockdown of PKM2 by siRNA triggers apoptosis in a panel of cancer cell lines, and substantially retards xenograft growth [34]. However, there are very few reports concerning the efficient delivery of siRNA against PKM2 to tumor cells via gene delivery systems [35,36,37]. In this work, we designed and synthesized a PEI-based siRNA delivery system by integration biodegradable polylactide (PLA) into branched PEI via a facile method, and utilized PEI-PLA copolymer for effective delivery of siRNA against PKM2 for the first time. The cytotoxicity of PEI-PLA copolymer against three human cancer cell lines and its ability to bind siRNA were determined. Moreover, we assessed the serum stability and cellular internalization of the formed polyplex. Finally, we exploited the effectiveness of this delivery system in restraining the expression of PKM2 in three cancer cell lines at both the mRNA and protein levels.

## 2. Materials and Methods

### 2.1. Materials

Branched PEI (Mw ~25 kDa, average Mn ~10 kDa) and poly(D,L-lactic acid) (Mw ~15 kDa) were obtained from Sigma-Aldrich and Polysciences Inc. (Germany), respectively. Acryloyl chloride was purchased from TCI (Shanghai) Development Co., Ltd. (Shanghai, China). Triethylamine was provided by Sigma-Aldrich. siRNA targeting PKM2 (siPKM2): 5′-CCAUAAUCGUCCUCACCAA-3′ (sense), and negative control siRNA (siNC) were synthesized by Sangon Biotech Co., Ltd. (Shanghai, China). Cy5-labled siRNA targeting PKM2 was provided by GenePharma Co. (Shanghai, China). Bicinchoninic acid (BCA) assay kit was obtained from Beyotime Institute of Biotechnology (Shanghai, China). Lipofectamine^®^ 2000 (Lipo 2000) Reagent was provided by Invitrogen Life Technologies (Carlsbad, CA, USA). Primary antibodies against PKM2 was purchased from Cell Signaling Technology (Danvers, MA, USA). All other organic solvents used were of analytical grade. HCT116, HepG2 and SKOV3 cells were obtained from Shanghai Cell Bank of Chinese Academy of Sciences (Shanghai, China).

### 2.2. Synthesis of Acrylated PLA

The acrylated PLA was synthesized according to a previous report [38]. Briefly, hydroxyl-ended PLA (0.5 mmol) was dissolved in 20 mL freshly distilled toluene, and acryloyl chloride (1.72 mmol) and triethylamine (1.72 mmol) were added. The mixture was gradually heated to 80 °C with magnetic stirring for 8 h and cooled to room temperature. The resultant solution was filtered to remove triethylamine hydrochloride and the filtrate was poured into cold n-hexane to precipitate the product, which was separated from the supernatant by decantation. Finally, the product was dried under vacuum at room temperature for 24 h.

### 2.3. Synthesis of PEI-PLA Copolymer

Acrylated PLA (1 g, ~0.044 mmol) and PEI (1 g, ~0.1 mmol) were dissolved in 5 mL DMSO respectively, and the two solutions were mixed and magnetically stirred. The Michael addition reaction was allowed to occur under a nitrogen atmosphere for 24 h. The solution was transferred to a dialysis bag (Mw cutoff: 8000−14000 Da) and dialyzed against high-purity water for 48 h. The resulting aqueous solution was lyophilized to obtain the product. To improve its water solubility, 10 mg PEI-PLA copolymer was dissolved in 0.5 mL DMSO, the polymer solution was slowly added to 10 mL of high-purity water under vigorous ultrasonic agitation. Next, the solution was dialyzed against water for 48 h to remove DMSO and lyophilized.

### 2.4. Determination of siRNA Complexation by Gel Retardation Assay

The complexation between siRNA and PEI-PLA copolymer was estimated using agarose gel electrophoresis. Nuclease-free water was used for siRNA complexation and the preparation of siRNA and polymer stock solutions. For preparation of polyplex with different N/P ratios (0.5, 1, 2, 3, 5, 7.5, 10, 12.5 and 20), different volume of PEI-PLA copolymer solution (100 μg/mL) and siRNA stock solution (5 μM) were mixed, diluted with nuclease-free water to a final volume of 10 μL, and incubated for 30 min at room temperature for polyplex formation. The binding of siRNA to PEI-PLA copolymer was determined by 1% agarose gel electrophoresis containing GelRed. Electrophoresis was performed at a constant voltage of 90 V for 35 min in TBE running buffer, and the retardation of siRNA mobility was visualized by irradiation with UV light.

### 2.5. Determination of the Serum Stability of siRNA Polyplex

The polyplex with an N/P ratio of 10 was used for serum stability study, and experiment was carried out as described in the literature [35,39]. The naked siRNA and siRNA polyplex with a N/P ratio of 10 were incubated with 10% or 50% serum at 37 °C for different time (0.5, 1, 2, 4, 8, 12, 24, 48 and 72 h), and immediately mixed with gel loading buffer with or without 1% SDS. All samples were analyzed by 1% agarose gel electrophoresis containing GelRed in TBE buffer, and the siRNA band was visualized.

### 2.6. Cytotoxicity of PEI-PLA Copolymer

The cytotoxicity of PEI and PEI-PLA copolymer was evaluated by MTT assay in three different cancer cell lines—human colon cancer cell line HCT116, human hepatocarcinoma cell line HepG2, and human ovarian cancer cell line SKOV3. The cells were seeded into 96-well plates at a density of 5000 cells per well, and incubated overnight. Different concentrations (0, 0.625, 1.25, 2.5, 5, 10 μM) of PEI and PEI-PLA copolymer were added into each well and the cells were incubated for 48 h. Then the medium in each well was removed and replaced by fresh medium, 20 μL MTT solution (5.0 mg/mL in PBS) were added per well, and the cells were incubated for another 4 h. Finally, the medium was removed, 150 μL DMSO was added to each well, and the absorbance at 570 nm was recorded with a microplate reader.

### 2.7. Cellular Internalization of siRNA Polyplex

HCT116, HepG2 and SKOV3 cells were seeded into 12-well plates with glass slides, and incubated overnight. Polyplex prepared with Cy5-labled siRNA, Cy5-labled siRNA transfected with Lipo 2000 (3 μL per well) and naked Cy5-labled siRNA with a siRNA concentration of 50 nM were added to each well and incubated for 12 h. After washing with PBS three times, cells were treated with 4% paraformaldehyde for 30 min and stained with DAPI for 10 min, then the distribution of siRNA inside the cells was observed by the Deltavision Elite Microscopy Imaging Systems (GE Healthcare) to evaluate the transfection efficacy.

### 2.8. Gene Silencing Efficiency In Vitro

#### 2.8.1. Real-Time PCR Assays for mRNA Level of PKM2 Gene

HCT116, HepG2 and SKOV3 cells were seeded into 6-well plates, incubated overnight and treated with polyplex, naked siRNA against PKM2 or transfected with Lipo 2000 (5 μL per well) for 144 h. siRNA transfection was repeated at 48 and 96 h to retain the knockdown of PKM2. Total RNA was extracted, reverse transcribed into cDNA and subjected to qPCR analysis on Bio-Rad CFX ConnectTM Real-Time System. GAPDH was used as an internal reference. The primer sequences used for PKM2 and GAPDH amplification were shown as following. PKM2 forward (GCTGCCATCTACCACTTGC), PKM2 reverse (CCAGACTTGGTGAGGACGATT); GAPDH forward (AAGGTCGGAGTCAACGGATTT), GAPDH reverse (CCTGGAAGATGGTGATGGGATT). Standard curves were established and the relative amount of PKM2 mRNA was normalized to mRNA levels of GAPDH.

#### 2.8.2. Western Blot Analysis of PKM2 Expression

HCT116, HepG2 and SKOV3 cells were seeded into 60 mm dish and exposed to polyplex, naked siRNA against PKM2 or transfected with Lipo 2000 (8 μL per dish) for 48 h. The cells were lysed and total protein was extracted and quantified. Protein samples were separated by 12% sodium dodecyl sulfate polacrylamine gel electrophoresis (SDS-PAGE) and then transferred to polyvinylidene difluoride (PVDF) membranes. The membranes were incubated overnight at 4 °C with rabbit antibody against PKM2 (1:1000 dilution, Cell Signaling Technology, Danvers, MA, USA) after blocking with 5% skim milk. Meanwhile, the membranes were incubated with rabbit antibody against GAPDH (1:1000 dilution; Cell Signaling Technology, Danvers, MA, USA) as an internal standard for normalization of protein levels.

### 2.9. Statistical Analysis

All quantitative data were expressed as mean ± standard deviation (SD). Statistical analysis was conducted using Student’s t-test, and differences were considered statistically significant when *P* < 0.05 (*) and highly significant when *P* < 0.01 (**).

## 3. Results and Discussion

### 3.1. Synthesis and Characterization of PEI-PLA Copolymer

**Figure 1:**
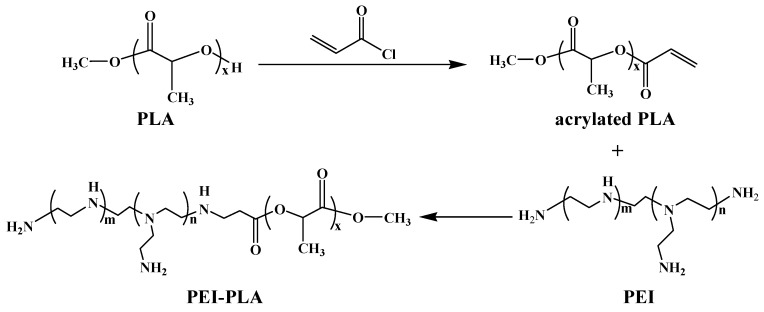
Synthetic scheme of PEI-PLA copolymer.

**Figure 2:**
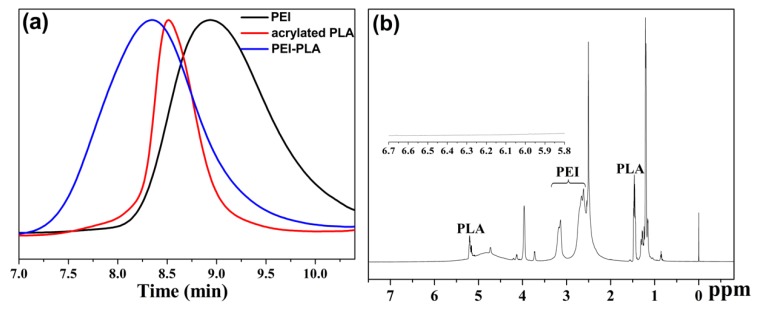
(**a**) GPC chromatograms of PEI, acrylated PLA and PEI-PLA copolymer; (**b**) ^1^H NMR spectrum of PEI-PLA copolymer in DMSO-d6, the inset represents magnification of 5.8−6.7 ppm.

The synthesis procedure of PEI-PLA copolymer was shown in Figure 1. Excess acryloyl chloride was used for complete acrylation of the terminal hydroxyl groups of polylactide (PLA), and the byproduct triethylamine hydrochloride could be easily removed by filtration. PEI-PLA copolymer was synthesized by conjugation of acrylated PLA and the primary amine group of PEI via Michael addition. Firstly, FTIR (Fourier transform infrared) spectroscopy was used for characterization of the synthesis of PEI-PLA copolymer. FTIR spectra of PEI, acrylated PLA and PEI-PLA copolymer in the range of 2000–1000 cm^−1^ are presented in Figure S1. The bands at 1594 cm^−1^ and 1332–1043 cm^−1^ are attributable to the -NH- bending vibration and C-N stretching vibration of primary and secondary amine groups of PEI, and the strong band at 1454 cm^−1^ is assigned to the bending vibration of -CH_2_- groups of PEI (Figure S1a). For acrylated PLA, the strong peaks at 1748 cm^−1^, 1183–1080 cm^−1^ and 957 cm^−1^ correspond to –C=O stretching vibration, -C-O- stretching vibration, and C=C absorption of acrylated PLA, respectively (Figure S1b). The characteristic peaks of both PEI (1594 cm^-1^, 1454 cm^-1^ and 1314–1033 cm^−1^) and acrylated PLA (1742 cm^−1^ and 1080 cm^−1^) can be detected in the spectrum of PEI-PLA copolymer (Figure S1c), and signal at 957 cm^−1^ from C=C of acrylated PLA disappeared, indicating the complete conversion of acrylated PLA to PEI-PLA copolymer. Next, gel permeation chromatography (GPC) was employed to determine the molecular weight and its distribution (polydispersity index, PDI) of PEI-PLA copolymers. As shown in Figure 2a and Table 1, the obtained PEI-PLA copolymer exhibits a unimodal molecular weight distribution as PEI and acrylated PLA. The molecular weight and PDI of PEI-PLA copolymer were 21,644 Da and 2.369, respectively, and it was eluted earlier (8.34 min) than PEI (8.99 min) and acrylated PLA (8.55 min), indicating that the PEI-PLA copolymer was obtained as expected. Moreover, the chemical structure of PEI, PLA-OH, acrylated PLA and PEI-PLA copolymer was analyzed by ^1^H NMR spectrometry (Figure S2 and Figure 2b). The signals of -CH_2_-CH_2_- groups and –NH- groups from PEI were observed in Figure S2a. Compared to the spectrum of PLA-OH (Figure S2b), the peaks from CH_2_=CH- groups can be detected in the inset of Figure S2c, suggesting the successful conjugation of acryloyl chloride with PLA-OH. The chemical signal of PEI and PLA could be seen in Figure 2b, meanwhile the CH_2_=CH- signal at 5.8–6.7 ppm disappeared, indicating the PEI-PLA copolymer was synthesized successfully.

### 3.2. Gel Retardation Assay of the PEI-PLA@siRNA Polyplex

**Figure 3:**
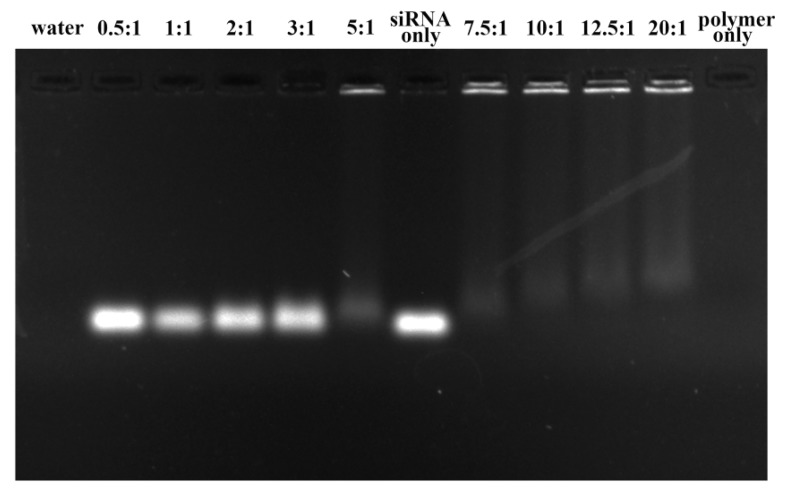
Agarose gel electrophoresis of PEI-PLA@siRNA polyplex at various *N*/*P* ratios.

The capacity of PEI-PLA copolymer to condense siRNA was monitored by gel retardation assay. Polyplex with different N/P ratios (0.5, 1, 2, 3, 5, 7.5, 10, 12.5 and 20) was prepared by incubation of different volume of PEI-PLA copolymer with siRNA for 30 min, and subjected to 1% agarose gel electrophoresis. It can be clearly seen from Figure 3 that the intensity of siRNA band gradually decreased along with the increase of N/P ratio, and completely disappeared when the N/P ratio is higher than 7.5. This result demonstrated that the mobility of siRNA was completely retarded at and above an N/P ratio of 7.5, indicating siRNA was bound tightly with PEI-PLA and no free siRNA could be observed. In addition, the polyplex prepared with an N/P ratio of 10 was employed in the following study.

### 3.3. Serum Stability of PEI-PLA@siRNA Polyplex

**Figure 4:**
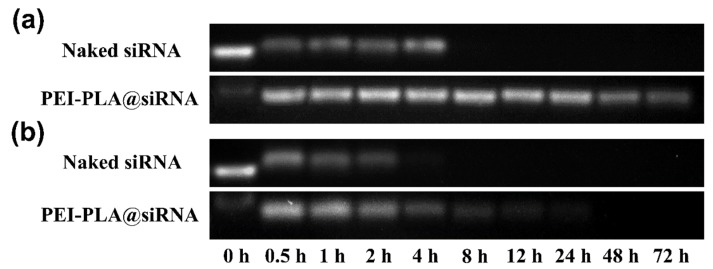
Agarose gel electrophoresis analysis of naked siRNA and PEI-PLA/siRNA polyplex after incubation with 10% (**a**) and 50% (**b**) serum for different time. siRNA can be dissociated from the polyplex in the presence of SDS. The naked siRNA and PEI-PLA/siRNA polyplex samples in the first lane (0 h) of (**a**) and (**b**) were prepared in the presence and absence of 1% SDS, respectively.

Naked siRNAs are susceptible to attack by serum nucleases, and the circulation stability of siRNA-based agents is a prerequisite for in vivo application [40]. Thus, the serum stability of naked siRNA and PEI-PLA@siRNA was determined by agarose gel electrophoresis followed by incubation with 10% and 50% serum at 37 °C for different time. siRNA could detach from the polyplex in the presence of SDS. It can be seen from Figure 4 that the interaction between PEI-PLA copolymer and siRNA was strong enough to withstand serum treatment, and we can see the siRNA band from polyplex after incubation in 10% and 50% serum for 72 h and 8 h, respectively. It is interesting that we cannot observe the siRNA band from PEI-PLA@siRNA polyplex at 0 h in the absence of SDS (Figure 4, first lane). By comparison, the band from naked siRNA cannot be detected after incubation in 10% serum for 4 h and in 50% serum for 2 h (Figure 4). These results demonstrated that the PEI-PLA@siRNA polyplex could markedly resist serum nucleases degradation and increase the stability of siRNA.

### 3.4. In Vitro Cytotoxicity of PEI-PLA Copolymer

**Figure 5:**
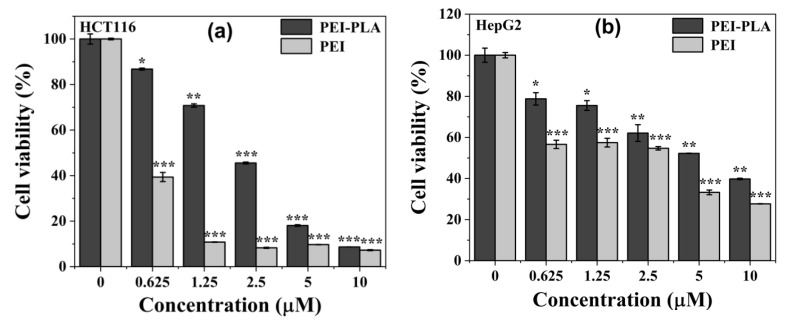

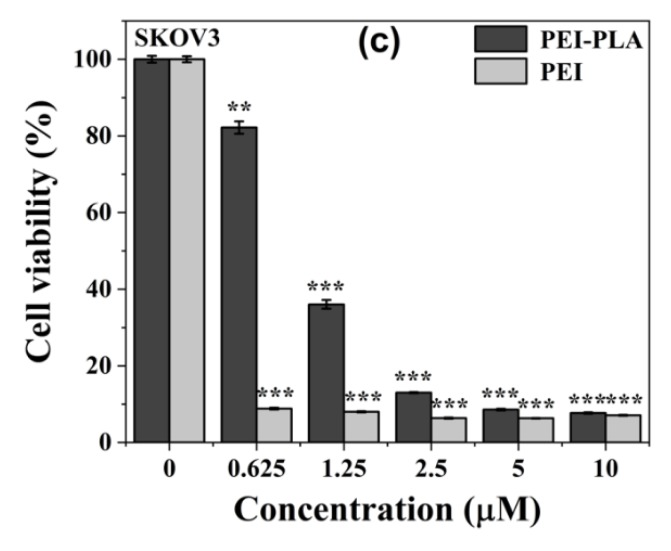
In vitro cytotoxicity of PEI and PEI-PLA copolymer at five different concentrations (0.625, 1.25, 2.5, 5 and 10 μM) to (**a**) HCT116, (**b**) HepG2 and **(c)** SKOV3 cells after 48 h treatment. * indicates *P* < 0.05, ** indicates *P* < 0.01, and *** indicates *P* < 0.001, relative to the control cells.

A versatile strategy for mitigating the toxicity of PEI is to introduce biodegradable polymers into PEI [19,41]. To explore whether the incorporation of biodegradable PLA improves the biocompatibility of PEI, we tested the cytotoxicity of PEI and PEI-PLA copolymer in three different cancer cell lines (HCT116, HepG2 and SKOV3) by MTT assay. As displayed in Figure 5, both PEI and PEI-PLA copolymer exhibited a dose-dependent cytotoxicity to the three tested cell lines. Interestingly, it is easy to find that integration of PLA into PEI obviously increased the percentage of cell viability of all three tested cells as compared to PEI (Figure 5). PEI-PLA copolymer also presented apparent cytotoxicity at higher concentrations due to the PEI component, and it shows the best biocompatibility to HepG2 cells (Figure 5b). It should be noted that the polyplex prepared with an N/P ratio of 10 was utilized in the following study, PEI-PLA copolymer concentration for polyplex preparation was 2.03 μM, and would be diluted to 0.0203 μM in the following gene silencing study with an siRNA concentration of 5 nM. Hence, the cytotoxicity of PEI-PLA would be negligible.

### 3.5. Intracellular Trafficking of PEI-PLA@siRNA Polyplex

**Figure 6:**
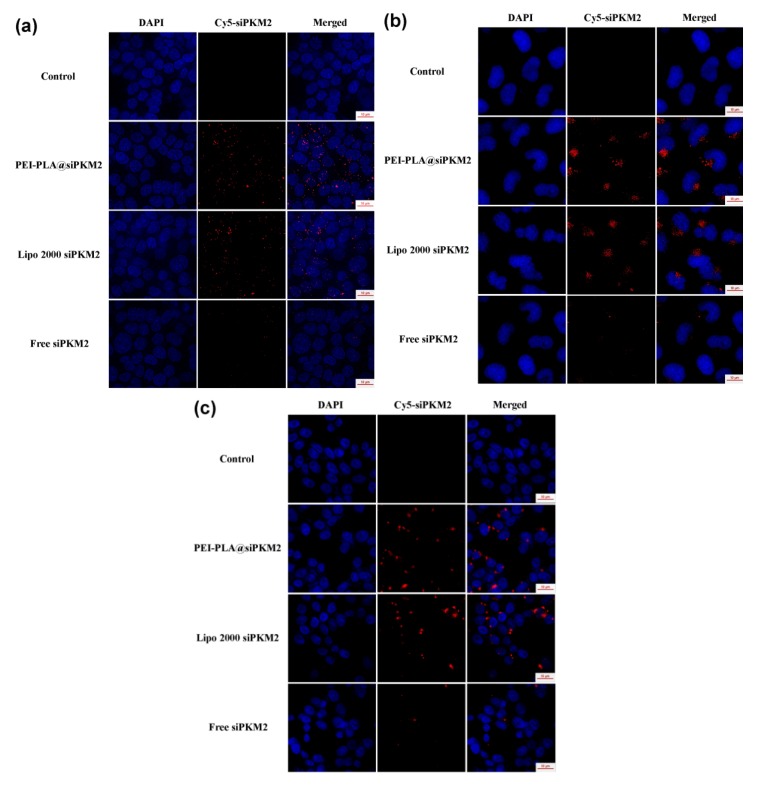
Representative images of of (**a**) HCT116, (**b**) HepG2 and (**c**) SKOV3 cells after transfection with PEI-PLA@siPKM2 polyplex, Lipo 2000 and free siPKM2 for 12 h. Cy5-labled siPKM2 was used in this experiment for visualization of cellular uptake and the nuclei were stained with DAPI.

A substantial obstacle to the therapeutic application of siRNAs is their inability to efficiently penetrate the cell membrane [42]. To gain insights into the cellular trafficking behavior of PEI-PLA@siRNA polyplex, Cy5-labled siPKM2 was employed to prepare polyplex, and its cellular uptake was examined in three cancer cell lines (HCT116, HepG2 and SKOV3). To evaluate and compare the internalization efficiency, the commercial transfection agent Lipo 2000 was also utilized for siPKM2 transfection, and free siPKM2 was used as control. As expected, we only observed several scattered red spots in free siPKM2-treated group in all three tested cell lines owing to the poor cellular uptake of naked siPKM2, while the blue DAPI signal from nuclei was observed (Figure 6). In contrast, we noticed extensive red fluorescence around the nuclei in PEI-PLA@siPKM2 and Lipo 2000 siPKM2 transfected groups, and there was little overlap of red and blue DAPI fluorescence (Figure 6), demonstrating that PEI-PLA copolymer and Lipo 2000 could deliver siPKM2 into the cytoplasm of all the three tested cell lines with high efficiency. Moreover, it is important to note that the transfection efficiency of PEI-PLA copolymer was equivalent to that of the commercial Lipo 2000. These results indicated that PEI-PLA copolymer had a great potential to be utilized as a versatile carrier for effective delivery of siRNAs into these three cancer cell lines.

### 3.6. Suppressive Effect of PEI-PLA@siRNA Polyplex on PKM2 Gene Expression

**Figure 7:**
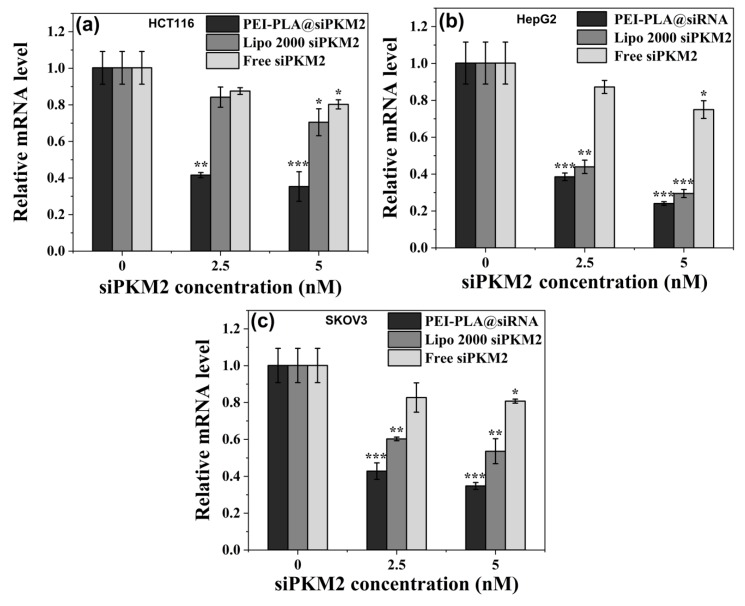
Real-time PCR analysis of PKM2 mRNA levels in HCT116 (**a**), HepG2 (**b**), and SKOV3 (**c**) cells. Cells were treated with PEI-PLA@siPKM2 polyplex, Lipo 2000 and free siPKM2 for 144 h, siRNA transfection was repeated at 48 and 96 h. * indicates *P* < 0.05, ** indicates *P* < 0.01, and *** indicates *P* < 0.001, relative to the control cells.

Given that PEI-PLA copolymer facilitated the cellular uptake of siPKM2 cargo, we further determined the gene knockdown efficiency of PEI-PLA@siPKM2 polyplex in three cancer cell lines at both mRNA and protein levels via RT-PCR and western blotting, respectively. As anticipated, exposure to PEI-PLA@siPKM2 polyplex and Lipo 2000 siPKM2 resulted in a noticeable and concentration-dependent reduction of PKM2 in mRNA level as compared to control in all three tested cell lines, and naked siPKM2 also lowered the level of PKM2 siRNA (Figure 7). Noteworthily, PEI-PLA copolymer displayed a stronger efficacy than Lipo 2000 in suppressing the PKM2 expression at mRNA level.

**Figure 8:**
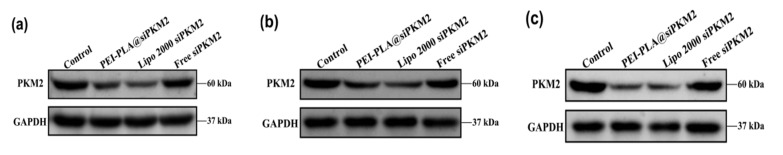

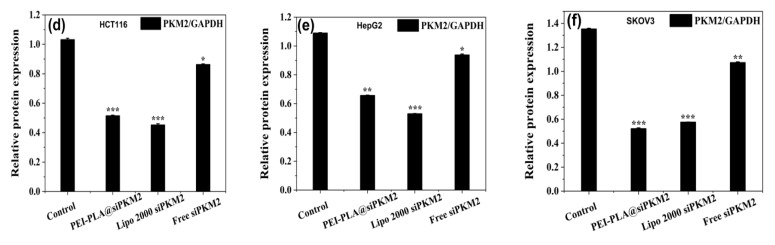
Western blot analysis of PKM2 protein expression in HCT116 (**a**), HepG2 (**b**), and SKOV3 (**c**) cells. GAPDH was used as an internal control. The relative protein expression levels in HCT116 (**d**), HepG2 (**e**), and SKOV3 (**f**) cells were quantified by densitometry. Cells were treated with PEI-PLA@siPKM2 polyplex, Lipo 2000 and free siPKM2 (siRNA concentration: 5 nM) for 48 h. * indicates *P* < 0.05, ** indicates *P* < 0.01, and *** indicates *P* < 0.001, relative to the control cells.

The expression of PKM2 at the protein level were further analyzed by western blotting. In line with the RT-PCR results (Figure 7), all three tested cell lines receiving PEI-PLA@siPKM2 polyplex and Lipo 2000 siPKM2 showed obviously lower level of PKM2 protein expression than control and free siPKM2-treated groups (Figure 8). PEI-PLA@siPKM2 polyplex and Lipo 2000 siPKM2 had similar inhibitory effect on PKM2 protein expression. Specifically, Lipo 2000 siPKM2 is slightly more potent than PEI-PLA@siPKM2 in HCT116 and HepG2 cells (Figure 8a,b,d,e), while PEI-PLA@siPKM2 has a little better performance in SKOV3 cells (Figure 8c,f). Consequently, these data suggested that PEI-PLA exhibited comparable efficiency to commercial Lipo 2000 in inhibiting the expression of PKM2.

## 4. Conclusions

In summary, we constructed an effective and safe siRNA delivery vector by integrating biocompatible PLA into branched PEI through a simple synthetic strategy. By combining the excellent transfection ability of PEI and the biodegradable property of PLA, the PEI-PLA copolymer showed much lower cytotoxicity than PEI to the three tested cancer cells while retaining the transfection capacity of PEI. The polymer-siRNA polyplex dramatically improved the stability of siRNA in serum, and could efficiently deliver the siRNA targeting PKM2 into the three cancer cells. Furthermore, it is encouraging to find that the polyplex is comparable to the commercial Lipo2000 in suppressing the expression of PKM2 at both mRNA and protein levels in three cancer cell lines. Taken together, the PEI-PLA copolymer synthesized in this study holds great promise to be developed as a transfection reagent and should be further explored with the goal of co-delivering antitumor agents and therapeutic genes.

## Figures and Tables

**Table 1:** The molecular weight, PDI and retention time of PEI, acrylated PLA and PEI-PLA copolymer as determined by GPC.

Sample	Molecular Weight (Da)	PDI	Retention Time (min)
PEI	5453	2.073	8.99
Acrylated PLA	14,253	1.300	8.55
PEI-PLA	21,644	2.369	8.34

## Supplementary Materials

The following are available online at https://www.mdpi.com/2073-4360/12/2/445/s1, Figure S1: FTIR spectra of PEI (a), acrylated PLA (b) and PEI-PLA copolymer (c); Figure S2: ^1^H NMR spectra of PEI (a), PLA-OH (b), acrylated PLA (c) in CDCl_3_. The inset in (b) and (c) represents magnification of 5.8−6.7 ppm, in which the CH_2_=CH- signal can be detected.
