# Supplementary material for: Integration of Polylactide into Polyethylenimine Facilitates the Safe and Effective Intracellular siRNA Delivery
Source: Polymers (Basel). 2020 Feb 14;12(2):445. doi: 10.3390/polym12020445 (PMC7077636; doi:10.3390/polym12020445)
Supplement: Supplementary file 1 [file polymers-12-00445-s001.pdf]

Supplementary Material

Integration of polylactide into polyethylenimine facilitates the safe and effective intracellular siRNA delivery

Guo-Bin Ding,[1,2,*] Xue Meng,[1,2] Peng Yang,[1,2] Binchun Li,[1] Roland H Stauber,[1,3] Zhuoyu Li [1,2,*]

[1]Institute of Biotechnology, the Key Laboratory of Chemical Biology and Molecular Engineering of Ministry of Education, Shanxi University, Taiyuan 030006, China

[2]Institutes of Biomedical Sciences, Shanxi University, Taiyuan 030006, China

[3]Molecular and Cellular Oncology, University Medical Center Mainz, Langenbeckstrasse 1, Mainz 55101, Germany

E-mail: dinggb2012@sxu.edu.cn (G.B. Ding); lzy@sxu.edu.cn (Z. Li)

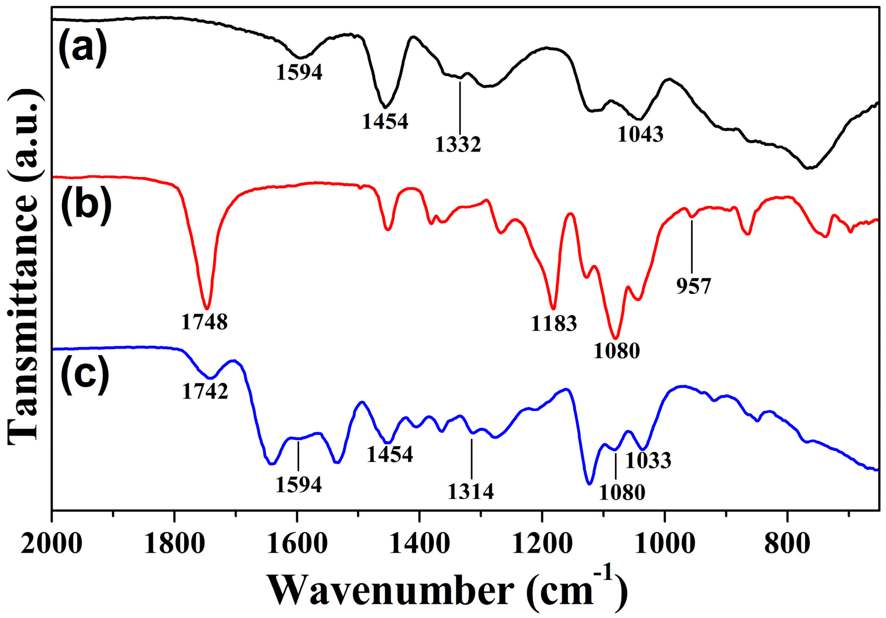

Figure S1. FTIR spectra of PEI (a), acrylated PLA (b) and PEI-PLA copolymer (c).

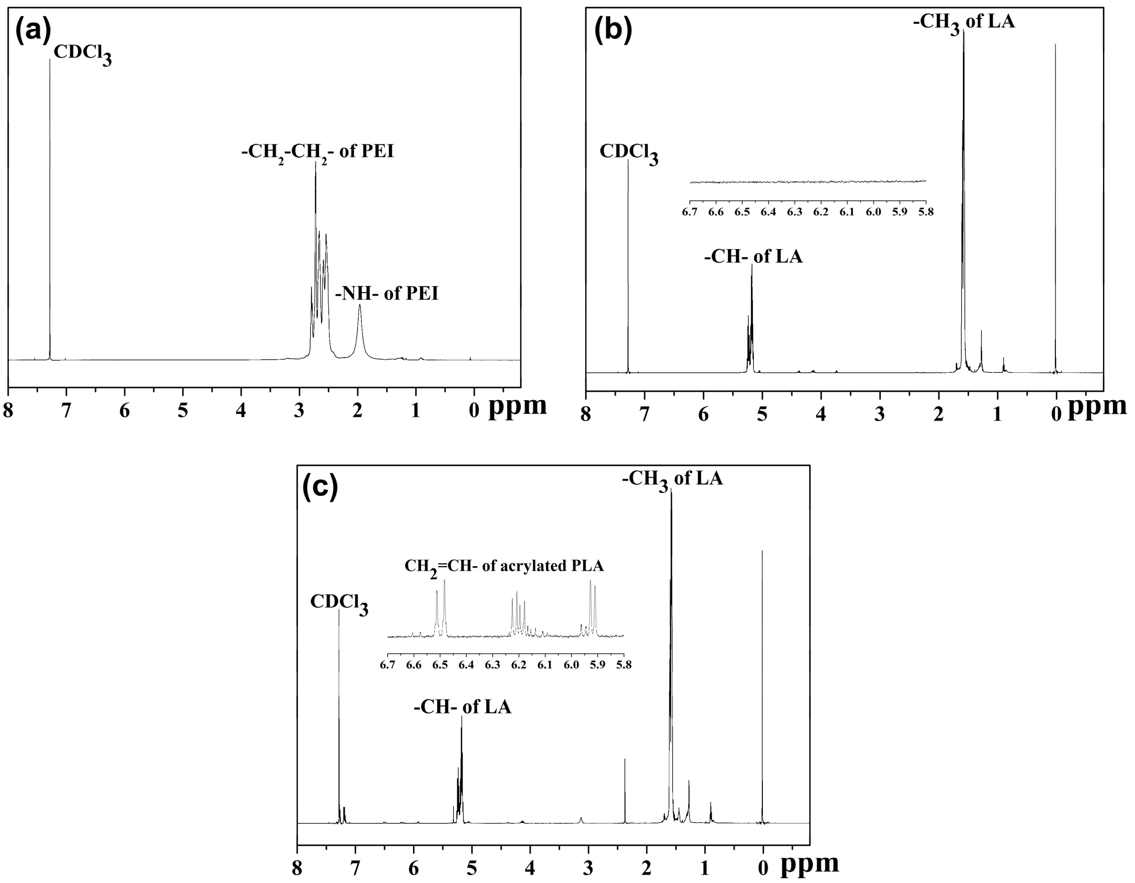

Figure S2. ^{1}H NMR spectra of PEI (a), PLA-OH (b), acrylated PLA (c) in $CDCl_3$. The inset in (b) and (c) represents magnification of 5.8−6.7 ppm, in which the CH_2=CH- signal can be detected.
